# Animal welfare during transport and slaughter: an issue that remains to be solved

**DOI:** 10.1093/af/vfac004

**Published:** 2022-03-17

**Authors:** E M Claudia Terlouw, Isabelle Veissier

**Affiliations:** Université Clermont Auvergne, INRAE, VetAgro Sup, UMR Herbivores, 63122 Saint-Genes-Champanelle, France

In the European Union (EU), in 2019, 7 billion animals—mainly cattle, sheep, pigs, and birds—were slaughtered for meat consumption (Food and Agriculture Organization of the United Nations [FAO], 2021). Meat and meat products are considered an excellent source of zinc, heme-iron, bioavailable B vitamins, and essential amino acids (De Smet and Vossen, 2016). The consumption of meat is, however, inherently related to animal transport and slaughter. For thousands of years, ethical questions have arisen relative to the killing of animals for consumption. Among the ancient Greek philosophers, Pythagoras (570–495 BCE) indicated: “As long as man continues to be the ruthless destroyer of lower living beings he will never know health or peace. For as long as men massacre animals, they will kill each other.” Theophrastus (371–287 BCE) thought that humans and animals have the same way of perception, reasoning, and appetites and condemned the consumption of meat, saying that killing animals is unjust because it robs them of life (Taylor, 2009). Porphyry (c.232–c.304) argued that we owe justice to animals not simply because they are rational but also because they are conscious beings who can feel pain and terror (Taylor, 2009). By contrast, Aristotle (384–322 BCE) thought that animals are entirely without reason and are ruled by their instincts, and that it is only proper that they should be used for human purposes (Taylor, 2009). Much later, in a similar way, Descartes (1596–1650) thought that animals are to be understood in purely mechanical terms. Although Descartes admits that animals have feelings of fear, hope, joy, anger, and hunger, he felt that we have good reason to believe they do not think. He wrote not denying “sensation to animals, insofar as it derives from a bodily organ (…). My view is not so much cruel to beasts but respectful to human beings (…), whom it absolves from any suspicion of crime whenever they kill or eat animals” (reprinted in Penguin Classics’ edition of *Meditations and Other Metaphysical Writings*, 2000; Descartes, 1999). 

Today, we have more, scientifically based, knowledge on these questions. Studies on behavior, physiology, and the anatomy and functioning of the brain indicate that it is likely that animals have emotional and cognitive capacities (Panksepp, 2005; Paul et al., 2005; Boissy et al., 2007). Negative and positive emotions help the animal to optimize its chances to survive and reproduce, that is, to adapt, because emotions are important drivers of motivation. Animals tend to avoid situations that result in negative emotions and are attracted to those that provide positive emotions. The origin of negative emotions may be psychological, such as fear, due to social disturbances, sudden events, and the unfamiliarity of the different situations the animals encounter. They may also be of physical origin, such as food deprivation or fatigue. Hence, both psychological and physical factors may influence the emotional status of the animal (Terlouw and Bourguet, 2022).

Despite this new knowledge, the debate on how animals must be treated is far from being closed, as the present Special Issue shows. Although most people agree that animals should be protected, many questions, relative to under which conditions and to which extent animals may be used by humans for consumption, for pleasure, or other objectives, remain.

Browning and Veit (2022) address the question of whether the moment in life where a positive or negative event occurs influences its valence. The authors indicate “… it may be objectively no worse for any harm to occur at some specific moment within a life rather than another – as objectively we have temporal neutrality*….*” However, they suggest that the negative valence of the transport and slaughter could have a relatively strong weighting because it happens at the end of the life of the animal: “it may not just be the number and intensity of experiences that matter, but the way they are distributed within the life.” For example, “individuals will experience some events differently, based on what has preceded them.” They suggest that “even when two lives contain an equal total sum of positive and negative experiences, it is still worse to have suffering at the end of life,” basing their argument on human preferences: “We simply prefer earlier pains to later ones, an upward trajectory to a downward one.” These considerations need reflection and may open the way to debates on how the animals experience time and on their capacity to integrate into a larger view the valences of events in the past, present, and possibly, the future. 

Bachelard (2022) addresses questions related to stress during transport and indicates that although regulations exist, there is much room and necessity for improvement. She underlines that it remains difficult to have sufficient controls and inspections to ensure that the regulations are respected. She mentions problems of overcrowding, improper ventilation, and injuries. She highlights that regulations admit very long journeys, especially during exports to third countries, and that they do not cover all farm animal species.

The papers by Francione (2022) and Porcher (2022) were prepared in the following way. They each received four questions which they answered (parts 1 and 2): What is animal welfare (definition)? Does animal welfare matter? If yes, why? if no, why not? Is it acceptable to eat animals? If yes, why? if no, why not? Do all animals deserve equal consideration in terms of animal welfare? Subsequently, we sent the writings of each one to the other so that the two authors could react to what the colleague had answered (parts 3 and 4). The authors did not know who the other person was, until the four questions were answered. Francione (2022) takes a strong stance relative to the consumption of animal products. He indicates that all animals suffer and that humans are omnivores and can get all the nutrition needed by consuming plants. He concludes, “we cannot justify eating animals or animal products in any situation in which we are not compelled to do so. When we choose to eat animal products, we are treating animals as things irrespective of what we otherwise say or believe.”

Similarly, Porcher (2022) advocates the respect and protection of animals. Differently from Francione (2022), she indicates that the consumption of meat products is not wrong, because “we are not similar to them” and that “not compassion, but respect is foundational.” She writes, “If one day we will learn more about the sensitivities of plants (…), inevitably, pulling carrots will seem as brutal to us as chopping down a tree. Then, unless we base our food resources on food biotechnology and cellular agriculture, we will have to resolve to starve in a very ethical way.”

Are other alternatives than plants possible? Chriki et al. (2022) indicate not only that meat could be replaced by artificially produced “cultured meat,” but also that “cultured meat” has very different properties from real meat. In addition, the killing of animals will still be necessary, because the starting cell lines are obtained either from muscle satellite cells from recently slaughtered animals or from an embryo or umbilical cord. Furthermore, today, fetal bovine serum taken from fetuses of pregnant cows at slaughter is used for the growth medium. Artificial serum would need to be developed, although the authors think that some might consider this unnatural. The authors feel that much work is needed to fulfill consumer expectations using “cultured meat” and that a useful first step would be to reduce food waste to “make more food available and reduce the carbon footprint.”

Conficoni et al. (2022) address ethical values related to meat consumption in the larger context of the Islamic Eid al-Adha feast in a small town in Italy, Rovigo. The case study shows that the event was initially celebrated under illegal and uncontrolled circumstances. “To guarantee freedom of worship and freedom to express their beliefs freely and independent of the state secular dictates,” efforts were made both by the participants and by the authorities to improve the conditions under which the celebration took place. To reduce animal stress, the number of people present at the slaughter procedure was limited and special adequate equipment was installed. The authors indicate that to make such progress possible, people must be considered as individuals, and not as “a homogeneous wall of anonymous people” and that “direct contact with different realities” and openness in the exchanges are essential.

People may have different views of what is the reality; sometimes they agree on the arguments but do not rank them similarly and still come to different conclusions. The articles illustrate the individual and cultural diversities in the attitudes and beliefs relative to animal suffering and the ethical issues related to meat consumption. Conceptual advances and research may help improve animal protection or find alternative solutions, and openness and empathy with animals and among humans are foundational to come closer to experiencing a shared reality.

## About the Authors

**Claudia Terlouw**, PhD, HDR, is a scientist working at INRAE (French National Research Institute for Agriculture, Food and Environment). She is a biologist specialized in behavior and physiology related to animal stress and started to work on abnormal behaviors such as schedule-induced polydipsia in rats and stereotypic behavior in sows. Since her appointment at INRAE in 1992, she studies the causes of stress during the slaughter period as well as the consequences, in terms of animal welfare and meat quality. She further studies the neurobiological mechanisms underlying the effects of stunning techniques and bleeding on brain functioning, particularly relative to the induction unconsciousness and death. She transfers the knowledge produced to the scientific world as well as to field workers.

**Isabelle Veissier**, DVM, PhD, is a research director from INRAE (French National Research Institute for Agriculture, Food and Environment). She initially qualified as a veterinarian. Her interest quickly turned to animal behavior as a way to enter their “inner world.” She studied the different facets of the behavior of cattle and sheep, always following the idea that their behavior informs us about the way they see the world around them, their social behaviors, the mother–young relationships (and their rupture), the learning capacities, the so-called abnormal behaviors, etc. At present, she investigates sickness behavior detected by sensor-based technologies. The ultimate goal of her work is to meet requirements for animal welfare and production concomitantly.
